# Paricalcitol improves cognitive dysfunction associated with chronic post-thoracotomy pain in rats

**DOI:** 10.1016/j.ynpai.2026.100238

**Published:** 2026-10-20

**Authors:** Ning Zheng, Yufeng Zhang, Cancan Cheng, Munle Chin, Wenxiang Qing, Qin Liao

**Affiliations:** a Department of Anesthesiology, The Third Xiangya Hospital of Central South University, Changsha, Hunan, China; b Department of Anesthesiology, The Third People's Hospital of Chengdu, Sichuan, China

**Keywords:** Vitamin D, Cognitive dysfunction, Chronic pain, Neuroinflammation, Gut microbiota

## Abstract

Although cognitive dysfunction associated with chronic pain has attracted growing attention, few studies have specifically focused on cognitive dysfunction induced by chronic post-thoracotomy pain (CPTP). Neuroinflammation is well recognized as a critical driver of chronic pain-related cognitive dysfunction, and vitamin D receptor (VDR) activation exerts potent anti-neuroinflammatory effects. However, whether the selective VDR agonist paricalcitol can ameliorate CPTP-induced cognitive dysfunction remains unclear. Here, we investigated the therapeutic effects of paricalcitol on CPTP-associated cognitive dysfunction and explored the underlying mechanisms. Rats were assigned to receive thoracotomy (to establish the CPTP model) or sham surgery as control. Paricalcitol or normal saline was intraperitoneally administered once daily for 5 days before surgery and once every other day for 28 days postoperatively. Mechanical pain threshold was evaluated using von Frey filaments. Locomotor activity, novel object recognition, and Barnes maze tests were performed to assess general locomotion and cognitive function. Hippocampal tissues were collected to detect key markers of neuroplasticity (doublecortin, NR1, NR2A, NR2B, GluA1, and GluA2) and neuroinflammation (IL-1β, TNF-α, and Iba1). Cecal contents were analyzed for gut microbiota composition, and colonic VDR protein expression was further quantified. We found that CPTP successfully induced persistent mechanical hypersensitivity and severe cognitive dysfunction, accompanied by excessive neuroinflammation, impaired neurogenesis, reduced NR2B expression, significant gut microbiota dysbiosis, and decreased colonic VDR protein levels. Notably, paricalcitol treatment substantially alleviated mechanical pain hypersensitivity and rescued CPTP-associated cognitive dysfunction. These beneficial effects were accompanied by improved hippocampal neuroplasticity, suppressed neuroinflammation, restored gut microbial homeostasis, and reversal of surgery-induced colonic VDR downregulation. Collectively, our findings demonstrate that paricalcitol ameliorates CPTP-associated cognitive dysfunction. These protective effects are associated with restored colonic VDR expression, suggesting a potential involvement of VDR signaling. This study provides a novel experimental basis for the potential clinical application of paricalcitol in preventing and treating pain-related cognitive dysfunction.

## Highlights

• Paricalcitol improves cognitive dysfunction associated with chronic post-thoracotomy pain.
• Paricalcitol increased hippocampal neuroplasticity-related molecules.
• Paricalcitol reduced hippocampal markers of neuroinflammation.
• Paricalcitol restored colonic VDR levels and altered gut microbiota composition.

## 1 Introduction

Chronic postsurgical pain is one of the most common complications following thoracotomy (Mauck et al., 2014), with incidence rates reaching up to 30–40% (Macrae, 2008). This specific form of chronic postsurgical pain, termed chronic post-thoracotomy pain (CPTP), is frequently accompanied by cognitive dysfunction. This comorbidity, namely cognitive dysfunction associated with CPTP, has attracted increasing attention owing to its profound impacts on patients' quality of life and healthcare burden (Liu et al., 2014). Clinical studies found that, compared to control individuals, patients with chronic pain have performed poorly on several parameters, including spatial (Luerding et al., 2008) and verbal (Weiner et al., 2006) working memory capacity, recognition memory (Park et al., 2001) and long-term spatial memory (Luerding et al., 2008). In animal experiments, impairments in spatial learning memory and behavioral flexibility were observed in a rat model of neuropathic pain (Hu et al., 2010). However, so far, these studies have primarily focused on neuropathic pain. Little attention has been paid to CPTP, despite its high incidence rate (Macrae, 2008). Additionally, little research has been conducted on cognitive dysfunction associated with CPTP. In this study, we aimed to investigate whether CPTP could induce cognitive dysfunction, marking the first exploration in this area.

Microglial activation and neuroinflammation induced by chronic pain have been reported in both humans and animal models. In one study using PET-MRI, patients with chronic low back pain experienced increased activation of microglia and astrocyte in the thalamus (Loggia et al., 2015). Animal models of chronic pain showed that the activation of microglia in the hippocampus (Dai et al., 2019), anterior cingulate cortex (Miyamoto et al., 2017), amygdala and other brain regions (Barcelon et al., 2019; Liu et al., 2017; Taylor et al., 2017). This pro-inflammatory state can lead to changes in synaptic remodeling, brain connectivity, and network function (Inoue and Tsuda, 2018), resulting in cognitive dysfunction (Bairamian et al., 2022).

Recently, several clinical and experimental findings have suggested a significant association between vitamin D deficiency and cognitive dysfunction (Ali et al., 2021; Mayne et al., 2019). Moreover, studies have indicated that vitamin D supplementation could improve working memory in mice (Morello et al., 2018). Higher serum 25 (OH)D levels were associated with a lower risk of dementia and AD in adult mice (Sakuma et al., 2019). Additionally, a study reported that low vitamin D levels were associated with poorer performance on cognitive tests in patients with Parkinson's disease (Santangelo et al., 2021). Vitamin D is an important hormon in regulating inflammation, immune responses via action of its receptor, the vitamin D receptor (VDR) (Shang and Sun, 2017). The inflammatory modulation properties of VDR indicate that it has the potential to ameliorate inflammation-associated cognitive dysfunction.

Paricalcitol, a vitamin D analogue, exerts anti-inflammatory effects via VDR activation (Lee et al., 2016). Unlike native vitamin D, paricalcitol functions as a selective VDR agonist that obviates the requirement for hepatic and renal bioactivation, enabling precise control over VDR-dependent transcriptional responses and exhibiting lower calcemic potential relative to calcitriol (Negrea, 2019). For these pharmacological merits, paricalcitol has been employed in multiple neuroinflammatory disease models, where it confers anti-inflammatory and neuroprotective activities (He et al., 2019; Zhang et al., 2020; Tian et al., 2026). Nevertheless, it remains unknown whether paricalcitol exerts beneficial effects on cognitive dysfunction associated with CPTP.

This study aimed to evaluate the protective efficacy of paricalcitol against CPTP-associated cognitive dysfunction in rats. We comprehensively assessed mechanical pain hypersensitivity and cognitive performance via behavioral tests. Hippocampal neuroplasticity markers and neuroinflammatory responses, including microglial activation and pro-inflammatory cytokine (IL-1β, TNF-α) expression, were measured to characterize central alterations induced by CPTP. We further analyzed gut microbiota composition and colonic VDR protein expression to delineate peripheral changes after thoracotomy and paricalcitol treatment. Together, these investigations help clarify the interplay among VDR expression, gut microbial homeostasis, neuroinflammation, and CPTP-related cognitive impairment.

## 2 Methods and materials

### 2.1 Animals

Eight-week-old male Sprague-Dawley (SD) rats were used in this study. The rats were housed in a quiet, uncrowded facility with a 12-h light/dark cycle (lights on 7 AM, off 7 PM) and ad libitum access to lab chow and water. The experiments were conducted in accordance with the guidelines of the National Institutes of Health on laboratory animal welfare and approved by the Animal Ethics Committee of the Central South University (The approval number: CSU-2022-0001-0312).

We anesthetized the rats using pentobarbital and 4.5% sevoflurane, and then intubated them. The endotracheal tube was connected to a small animal ventilator for mechanical ventilation (volume-controlled mode, tidal volume: 2.3 ml, respiratory rate: 80 breaths/min). Anesthesia was maintained with 2%–2.5% sevoflurane. Vital signs were monitored during the surgery.

After completion of the experiment, the rats were subjected to rapid- euthanasia by a single intraperitoneal injection of sodium pentobarbital at a dose of 100 mg/kg.

### 2.2 Experimental groups

#### 2.2.1 Experimental schematic

The detailed experimental flowchart was shown in Fig. 1A.

**Fig. 1:**
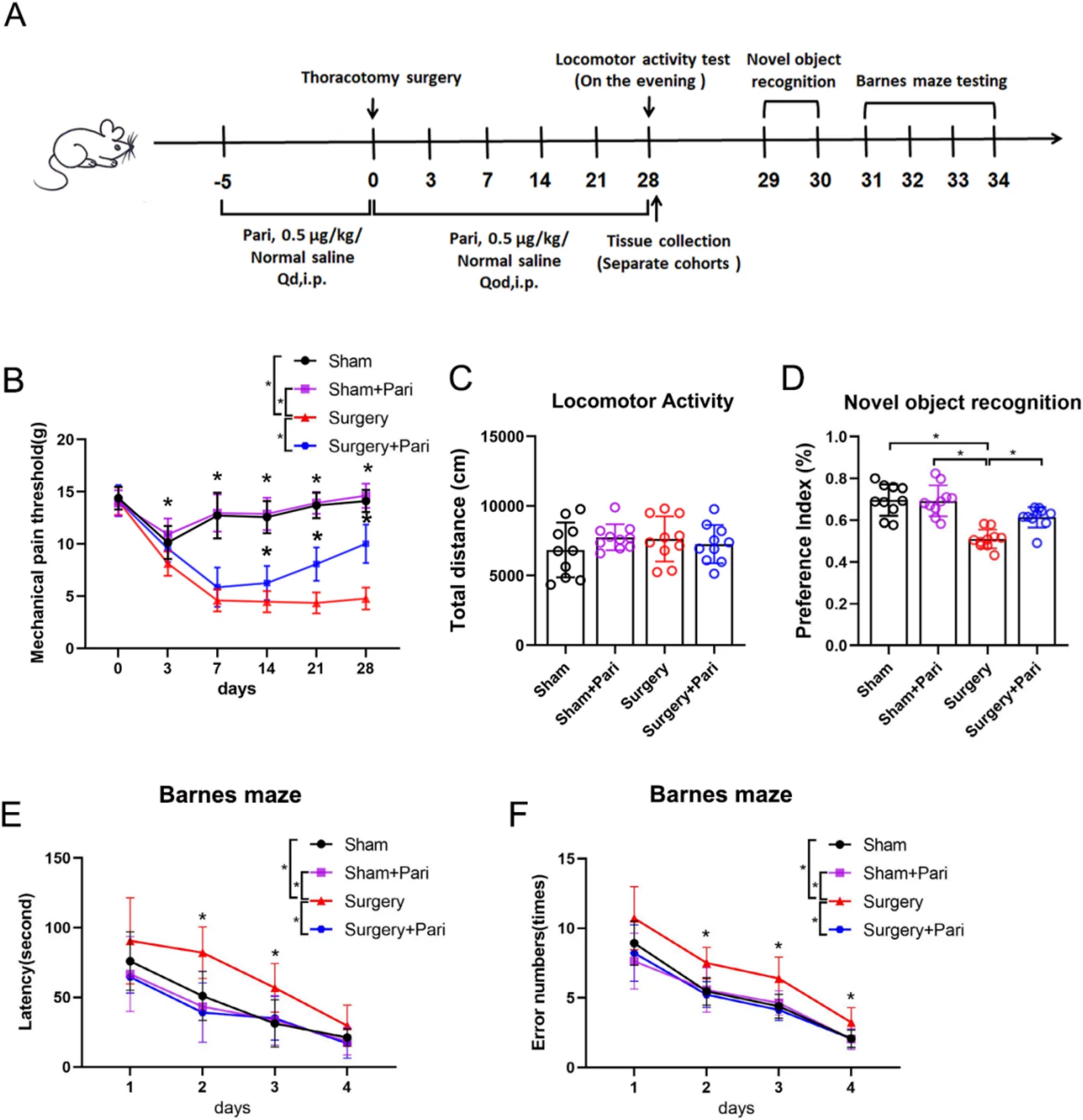
Paricalcitol alleviated chronic post-thoracotomy pain and the associated cognitive dysfunction. (A) Experimental schedule. (B) Mechanical pain thresholds in Sham, Sham+Pari, Surgery and Surgery+Pari. (C) Total distance in the locomotor activity test. (D) Preference index in the novel object recognition test. (E) Latency in the Barnes maze test. (F) Error numbers in the Barnes maze test. Data are presented as mean ± SD. ∗*p* < 0.05 for inter-group comparison in B-F. In B-F, post-hoc comparisons are between Surgery and each of the other three groups. *n* = 10 rats per group.

Paricalcitol or normal saline were injected intraperitoneally once daily for 5 days preoperatively, then once every other day for 28 days postoperatively. Mechanical pain thresholds were measured before surgery and on postoperative days 3, 7, 14, 21 and 28. Separate cohorts were used for tissue collection and behavioral testing. Hippocampal tissue, colonic tissues and cecal contents were collected on postoperative day 28 after pain-threshold testing. The locomotor activity test was performed on the evening of postoperative day 28, novel object recognition on days 29–30, and Barnes maze test on days 31–34.

#### 2.2.2 Experimental groups

The rats were randomly divided into four groups:

Sham group: Normal saline (volume-matched to paricalcitol) was injected intraperitoneally once daily for 5 days preoperatively, followed by sham surgery on day 0. Postoperatively, intraperitoneal normal saline was given once every other day for 28 days.

Sham+Pari group: Paricalcitol 0.5 μg/(kg.day) was injected intraperitoneally once daily for 5 days preoperatively, followed by sham surgery on day 0. Postoperatively, intraperitoneal paricalcitol 0.5 μg/(kg.day) was given once every other day for 28 days.

Surgery group: Normal saline (volume-matched to paricalcitol) was injected intraperitoneally once daily for 5 days preoperatively, followed by thoracotomy on day 0. Postoperatively, intraperitoneal normal saline was given once every other day for 28 days.

Surgery+Pari group: Paricalcitol 0.5 μg/(kg.day) was injected intraperitoneally once daily for 5 days preoperatively, followed by thoracotomy on day 0. Postoperatively, intraperitoneal paricalcitol 0.5 μg/(kg.day) was given once every other day for 28 days.

### 2.3 Thoracotomy

Prior to surgery, all instruments were autoclaved or sterilized with Cidex. The rats were anesthetized by intraperitoneal injection of pentobarbital sodium (60 mg/kg, Sigma-Aldrich Chemical Company, St. Louis, MO) and received thoracotomy in accordance with the previous studies (Zhu et al., 2018). Following endotracheal intubation with a 16-gauge catheter, rats were mechanically ventilated with a small animal volume controlled ventilator. The superficial and deep muscle layers were bluntly separated with a 2 cm incision made along the right 4–5 intercostal lines. Then an incision of 1.5 cm was then made along the cranial border of the fifth rib in the pleura and intercostal muscle. Using a blunt microscopic retractor, the ribs were spaced 1 cm apart and held for 60 min. During this time, the incision was covered with wet gauze. In order to eliminate intrathoracic air accumulation and restore intrathoracic negative pressure, a 22 g catheter was placed in the intrathoracic cavity before the chest wall was sutured with 3–0 chromic catgut. Suture the superficial muscles covering the ribs with 3–0 catgut and the skin with 3–0 nylon. After the recovery of spontaneous respiration, the endotracheal tubes were removed. In the sham group, the skin and pleural incision were performed without thoracic retraction.

### 2.4 Pain behavior test

Mechanical pain thresholds: According to the method described by Wang et al. (2013), a set of calibrated von Frey hairs (VFHs) was applied vertically to the skin over the surgical incision. The VFHs provided bending forces ranging from 0.4 g to 15 g. To determine the nocifensive response threshold, forces were applied in ascending order, starting at the contralateral side of the incision and moving to the ipsilateral side. A positive nocifensive response was defined as rat avoidance or scratching behavior evoked within 6 s of VFH stimulation. A maximum stimulus force of 15.0 g was used to prevent tactile sensitization. The classical “up-and-down” method was applied to calculate the corrected mechanical allodynia threshold for each rat. Data were then subjected to statistical analysis and visualized graphically.

### 2.5 Locomotor activity test

Locomotor activity was assessed in a 1 m × 1 m arena using video tracking (Seibenhener and Wooten, 2015). A digital camera recorded rat locomotor behaviors for 5 min while animals were placed in the central area of the apparatus. Total distance traveled by each animal served as the primary measure of horizontal locomotor activity. The open-field chamber was cleaned with 75% ethanol between tests. Only total distance traveled was included in the analysis; center and peripheral dwell times were not reported, and no inferences regarding anxiety-like behavior were drawn from this test.

### 2.6 Novel object recognition test

To conduct the novel object recognition test, we employed a 1 m × 1 m × 40 cm field arena. The protocol employed for the object recognition test was previously described in studies (Qing et al., 2018). The test consisted of both a training phase and a testing phase. During the training phase, we positioned two identical objects at equal distances from the center and walls of the arena. The rats were placed in the arena with their heads facing away from the objects and allowed to freely explore them for 10 min. Subsequently, during the testing phase, the rats were given a 10-min opportunity to explore the arena containing both familiar and novel objects. To maintain hygiene and eliminate potential bias, the objects and the chamber were thoroughly cleaned using a 75% alcohol solution between tests. The test capitalizes on rodents' innate tendency to investigate novel objects more extensively than familiar ones. By evaluating the time spent exploring novel objects, we can gauge their learning and recognition memory. Specifically, we calculated the preference index using the formula: novel object investigation time / (novel object investigation time + familiar object investigation time).

### 2.7 Barnes maze test

In animals studies, the Barnes maze is often used to evaluate hippocampus-dependent spatial learning and memory (Wu et al., 2020). The maze consisted of a platform 122 cm in diameter, elevated 140 cm above the ground, with 20 evenly spaced holes of 5 cm diameter. One hole was connected to a dark chamber called the target box, which served as an escape from the bright, aversive 200-watt light for the rats. Initially, rats were placed in a black cylinder positioned at the maze center for 15 s. The cylinder was then removed, and the rats explored the maze until locating the target box. If rats failed to find the target within 3 min, they were gently guided into the target box. Three trials were performed per day, with a 15-min interval between trials. Rats were permitted to stay in the target box for 1 min before being returned to their home cages. The Barnes maze was wiped with 75% alcohol between trials to eliminate odor cues. Escape latency and the number of incorrect hole visits (defined as errors) were recorded for each trial.

### 2.8 Real-time quantitative polymerase chain reaction (RT-qPCR) assay

RNA was isolated using TRIzol Reagent (Invitrogen, USA) and reverse transcribed following the manufacturer's instructions (Vazyme, China). RT-qPCR was performed using an mRNA qPCR kit from Vazyme (China). Primer sequences for the genes of interest were listed in Table 1.

**Table 1:** Primers used for real-time quantitative PCR.

Target gene	Primer	Sequence (5′-3′)
NR1	Forward	CAATGACCCCAGGCTCAGAA
	Reverse	GCTGCGCTCTCGTAATTGTG
NR2A	Forward	CATCCACGGCTTGGTGTTTG
	Reverse	CGTGGATGTCGGATCCTTGT
NR2B	Forward	TTGCCATCATCACCACTGCT
	Reverse	CCTTCCATTTCCCCACCCTC
GluA1	Forward	ACCGGATGCTCTTTCAGGAC
	Reverse	TCAATGTCCATGAAGCCCAGA
GluA2	Forward	TGCTTGAAGGGAATGAGCGT
	Reverse	TGCAATGTCAGCTTTCCCGT
TNF-α	Forward	ATGGGCTCCCTCTCATCAGT
	Reverse	GCTTGGTGGTTTGCTACGAC
IL-1β	Forward	GCTTCCTTGTGCAAGTGTCTG
	Reverse	CTCCACG GGCAAGACATAGG
GAPDH	Forward	TTGTGCAGTGCCAGCCTC
	Reverse	GATGGTGATGGGTTTCCCGT

### 2.9 Immunofluorescence analysis

Rats were anesthetized via sevoflurane inhalation and subjected to transcardial perfusion with 0.01 M PBS to flush out intravascular blood. Subsequently, 4% paraformaldehyde (PFA) was perfused for tissue fixation. Brains were harvested and post-fixed in 4% paraformaldehyde at 4 °C for 24 h. The tissues were sequentially cryoprotected in 15% and 30% sucrose solutions prepared in 0.01 M PBS at 4 °C. After cryoprotection, brains were embedded in OCT compound, and 20 μm coronal sections were serially cut using a frozen sliding microtome (Leica CM1950, Wetzlar, Germany). For immunofluorescent staining of ionized calcium-binding adapter molecule 1 (Iba1) and doublecortin (DCX), free-floating hippocampal sections were first washed with PBS, permeabilized with 0.5% Triton X-100 for 15 min, and blocked with 5% skim milk at 37 °C for 1 h. Sections were then incubated with primary antibodies against Iba1 (11,000, Wako, Japan) and DCX (11,000, Cell Signaling Technology, Danvers, USA). Following overnight incubation at 4 °C, sections were incubated with the corresponding secondary antibody (12,000, Beyotime) at room temperature for 1 h. Nuclei were counterstained with 4′,6-diamidino-2-phenylindole (DAPI), and fluorescence images were acquired using a fluorescence microscope (Nikon, Tokyo, Japan). Fluorescence intensity of DCX and Iba1 in the dentate gyrus was quantified. Three coronal sections were analyzed per rat (*n* = 4 rats per group) to generate a single fluorescence intensity value for each animal. Individual values were normalized to the mean fluorescence intensity of the control group, which was set to 1. Statistical analyses were performed using these normalized values.

### 2.10 Western blot

Tissues were homogenized in NP40 buffer containing 1% protease inhibitors and 1% phosphatase inhibitors (Sigma-Aldrich Co., MO, United States). The homogenate was then centrifuged at 12,000*g* for 20 min at 4 °C, and the supernatant was collected. The protein concentration was determined using the bicinchoninic acid (BCA) protein assay kit (CWBio, China).

Equal amounts of protein were separated by SDS-PAGE electrophoresis and transferred to the PVDF membranes. The membranes were blocked and subsequently incubated with the corresponding primary antibodies, anti-VDR (ab3508, Abcam, MA, United States), and anti-GAPDH (ab181603, Abcam, MA, United States), respectively. Afterward, they were incubated for an additional hour with the IRDye 800CW goat anti-rabbit secondary antibody (926–32211, LI-COR, United States). Immunoblotting bands were visualized using the Odyssey CLx infrared imaging system (LI-COR, United States). The protein levels were quantified by densitometry using ImageJ software (National Institutes of Health, MD, United States) and normalized to GAPDH levels.

### 2.11 Gut microbiota analysis

DNA was extracted from 200 mg of cecal contents using the QIAamp DNA Stool Mini Kit (QIAGEN, Hilden, Germany) per the manufacturer's protocol. Extracted DNA was stored at −20 °C for subsequent analysis. The V3–V4 region of the bacterial 16S rRNA gene was amplified using universal primers 338F (5’-ACTCCTACGGGAGGCAGCAG-3′) and 806R (5’-GGACTACHVGGGTWTCTAAT-3′). PCR amplicons were verified by electrophoresis on 1% agarose gels and purified with the Agencourt AMPure XP kit (Beckman Coulter, Brea, CA, USA). Sequencing was carried out on the Illumina MiSeq PE300 platform (Illumina Inc., San Diego, CA, USA). Alpha diversity metrics, including the Shannon and Chao indices, were calculated using the R package *vegan*. Partial Least Squares Discriminant Analysis (PLS-DA) was performed to discriminate the overall microbiota composition between groups. Bray–Curtis dissimilarity was used to assess β-diversity. The LEfSe algorithm was applied to identify differentially abundant taxa across groups at various taxonomic levels. The Kruskal–Wallis test with Bonferroni correction was used for pairwise comparisons to detect significant differences.

### 2.12 Statistical analysis

Data were analyzed using SPSS 18.0 (SPSS Inc., Chicago, IL, USA) and are presented as the mean ± standard deviation (SD). Mechanical pain thresholds and Barnes maze data were analyzed using repeated-measures analysis of variance (ANOVA). The remaining datasets were analyzed by one-way ANOVA followed by Tukey's post-hoc multiple comparison test. *P* < 0.05 was considered statistically significant.

## 3 Results

### 3.1 Paricalcitol alleviated chronic post-thoracotomy pain and the associated cognitive dysfunction

We first measured mechanical pain threshold in rats after thoracic surgery. Before surgery (day 0), mechanical pain thresholds did not differ significantly among the four groups. Two-way repeated-measures ANOVA revealed significant main effects of group [F (3, 36) = 238.0, *p* < 0.0001] and time [F(3.923, 141.2) = 76.77, *p* < 0.0001], as well as a significant group × time interaction [F(15, 180) = 23.44, *p* < 0.0001]. Tukey's post-hoc comparisons of group means averaged across all assessed time points showed that mechanical pain thresholds were significantly higher in the Sham and Sham+Pari groups than in the Surgery group (both *p* < 0.0001). The Surgery+Pari group also exhibited higher mechanical pain thresholds than the Surgery group (*p* = 0.0031), indicating attenuated mechanical hypersensitivity following paricalcitol treatment. At individual time points, mechanical pain thresholds were significantly lower in the Surgery group than in the Sham group on postoperative day 3 (*p* = 0.0223) and days 7, 14, 21, and 28 (all *p* < 0.0001). Compared with the Surgery group, the Surgery+Pari group exhibited significantly higher thresholds on postoperative day 14 (*p* = 0.0451) and days 21 and 28 (both *p* < 0.0001), whereas no significant differences were observed on days 3 or 7 (adjusted *p* = 0.0733 and 0.2859, respectively). The Sham and Sham+Pari groups did not differ significantly at any of these postoperative time points (all *p* > 0.05) (Fig. 1B).

Then, we assessed whether CPTP could induce cognitive dysfunction and evaluated the therapeutic efficacy of paricalcitol using locomotor activity test, novel object recognition test, and Barnes maze test. In the locomotor activity test, there was no significant difference in total travel distance among groups of Sham, Sham+Pari, Surgery and Surgery+Pari [Fig. 1C, one-way ANOVA, F (3, 36) = 0.7287, *p* = 0.5416]. In the novel object recognition test, the analysis of variance revealed a significant effect of group [Fig. 1D, one-way ANOVA, F (3, 36) = 19.78, *p* < 0.0001]. Post hoc analyses confirmed that the novel object preference index of the Surgery group was significantly less than that of the other three groups (Surgery vs. Sham: *p* < 0.0001, Surgery vs. Sham+Pari: *p* < 0.0001, Surgery vs. Surgery+Pari: *p* = 0.0034). In the Barnes maze test, the repeated measures ANOVA showed significant difference in latency and error numbers among groups [Fig. 1E: F (3, 144) = 16.19, *p* < 0.0001, Fig. 1F: F (3, 144) = 21.43, *p* < 0.0001]. Post hoc analyses of main effect showed that the Surgery group had significantly longer latency and higher error numbers compared to the other three groups. (Fig. 1E: Surgery vs. Sham: *p* < 0.0001, Surgery vs. Sham+Pari: *p* < 0.0001, Surgery vs. Surgery+Pari: *p* < 0.0001; Fig. 1F: Surgery vs. Sham: *p* < 0.0001, Surgery vs. Sham+Pari: *p* < 0.0001, Surgery vs. Surgery+Pari: *p* < 0.0001). Specifically, post hoc analyses of simple effect showed that the latency made by the Surgery group were significantly more than those made by the other three groups on day 2 and day 3 (Fig. 1E, day 2: Surgery vs. Sham: *p* = 0.006, Surgery vs. Sham+Pari: *p* = 0.0064, Surgery vs. Surgery+Pari: *p* = 0.0007; day 3: Surgery vs. Sham: *p* = 0.0183, Surgery vs. Sham+Pari: *p* = 0.0383, Surgery vs. Surgery+Pari: *p* = 0.0403). While the error numbers made by the Surgery group were significantly more than those of the other three groups on day 2, day 3 and day 4 (Fig. 1F, day 2: Surgery vs. Sham: *p* = 0.0025, Surgery vs. Sham+Pari: *p* = 0.0297, Surgery vs. Surgery+Pari: *p* = 0.0007; day 3: Surgery vs. Sham: *p* = 0.0133, Surgery vs. Sham+Pari: *p* = 0.0298, Surgery vs. Surgery+Pari: *p* = 0.0049; day 4: Surgery vs. Sham: *p* = 0.0435, Surgery vs. Sham+Pari: *p* = 0.0447, Surgery vs. Surgery+Pari: *p* = 0.0421). These results suggested that CPTP could induce cognitive dysfunction and paricalcitol could alleviate CPTP-associated cognitive dysfunction.

### 3.2 Paricalcitol rescued the downregulation of neuroplasticity-related molecules in CPTP rats

It has been shown that individuals with cognitive impairment are susceptible to impaired neurogenesis, NMDA receptors and AMPA receptors (Leiter et al., 2022). Thus, we detected the immature neuron marker Doublecortin (DCX), and the expression of AMPA receptor subunits (*GluA1* and *GluA2*), NMDA receptor subunits (*NR1*, *NR2 A*, and *NR2B*) among groups. There were significant differences in DCX intensity of fluorescence among groups [Fig. 2A and B, one-way ANOVA, F (; 3, 12) = 22.30, *p* < 0.0001]. Post hoc analyses showed that DCX intensity of fluorescence in the Surgery group significantly decreased (Fig. 2B, Surgery vs. Sham: *p* = 0.0001, Surgery vs. Sham+Pari: *p* < 0.0001). The DCX intensity of fluorescence in Surgery+Pari group was significantly more than that in the Surgery group (*p* = 0.0078). In addition, we found that the numbers of *NR2B* differed significantly among groups [Fig. 2G, one-way ANOVA, F (3, 20) = 6.918, *p* = 0.0022]. Significant downregulation of *NR2B* was detected in Surgery rats (Surgery vs. Sham:*p* = 0.028, Surgery vs. Sham+Pari: *p* = 0.0018). The numbers of *NR2B* in Surgery+Pari group was significantly more than that in the Surgery group (*p* = 0.0160). No obvious difference in *GluA1*, *GluA2*, *NR1* and *NR2A* was detected among the four groups [one-way ANOVA, Fig. 2C: F (3, 20) = 0.2719, *p* = 0.8449; Fig. 2D: F (3, 20) = 0.07054, *p* = 0.9750; Fig. 2E: F (3, 20) = 0.7736, *p* = 0.5224; Fig. 2F: F (3. 20) = 1.116, *p* = 0.3661].

**Fig. 2:**
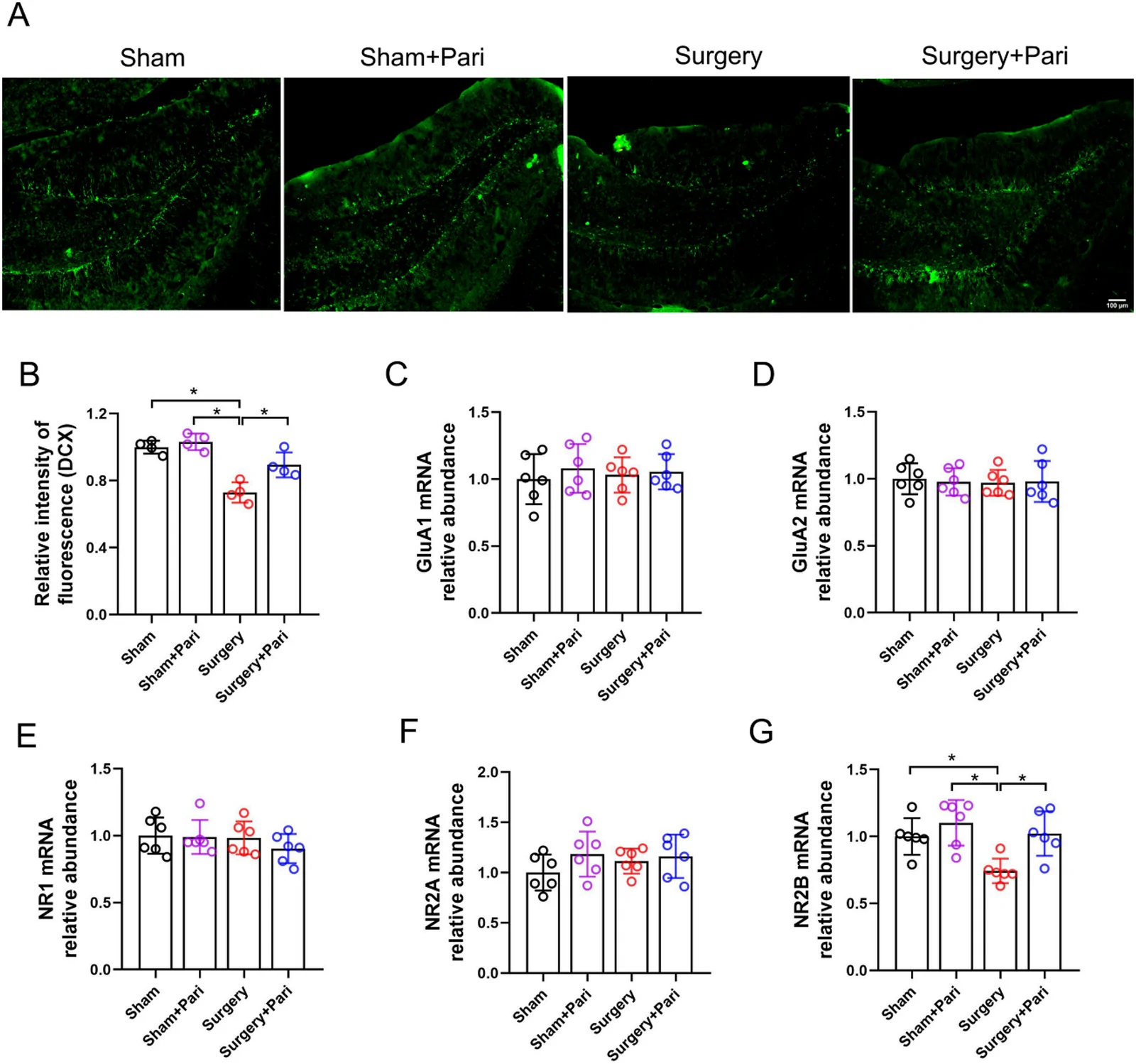
Paricalcitol rescued the downregulation of neuroplasticity-related molecules in CPTP rats. (A) Representative images of DCX staining in the dentate gyrus (DG). Scale bar = 100 μm. (B) Relative fluorescence intensity of DCX, *n* = 4 rats per group. (C) *GluA1* mRNA relative abundance. (D) *GluA2* mRNA relative abundance. (E) *NR1* mRNA relative abundance. (F) *NR2 A* mRNA relative abundance. (G) *NR2B* mRNA relative abundance. *n* = 6 rats per group for panels C-G. Data are expressed as the mean ± SD. *p < 0.05 for Surgery versus Sham, Sham+Pari or Surgery+Pari in B and G.

### 3.3 Paricalcitol inhibited hippocampal neuroinflammation in CPTP rats

Neuroinflammation contributes to cognitive dysfunction under multiple pathological conditions. Activated microglia serve as a key cellular marker for neuroinflammation (Chen et al., 2021). We therefore examined the microglial marker Iba1 in the hippocampus. In the present study, there were significant differences among groups based on Iba1 intensity of fluorescence (Fig. 3A and B, one-way ANOVA, *F* (3, 12) = 10.81, *p* = 0.0010). Hippocampal Iba1 fluorescence intensity was significantly higher in Surgery rats compared with Sham (*p* = 0.0022) and Sham+Pari rats (*p* = 0.0014). While Surgery+Pari partly reversed this phenomenon (*p* = 0.0385). Microglial activation drives the production of pro-inflammatory cytokines, including IL-1β and TNF-α (Liu et al., 2020). Therefore, we detected the expression of *IL-1β* and *TNF-α* in the hippocampus by RT-PCR. There were significant differences in the expression of *IL-1β* and *TNF-α* among groups [One-way ANOVA, Fig. 3C: *F* (3, 20) = 5.613, *p* = 0.0059, Fig. 3D: *F* (3, 20) = 8.698, *p* = 0.0007]. Post hoc analyses showed that the expression of *IL-1β* and *TNF-α* genes in Surgery rats were significantly upregulated, compared with those in groups Sham (*IL-1β*: *p* = 0.0104, *TNF-α*: *p* = 0.0015), Sham+Pari (*IL-1β*: *p* = 0.0111, *TNF-α*: *p* = 0.0052) and Surgery+Pari (*IL-1β*: *p* = 0.0470, *TNF-α*: *p* = 0.0022). These results demonstrated that CPTP-associated cognitive dysfunction was accompanied by hippocampal neuroinflammation, which was attenuated by paricalcitol treatment.

**Fig. 3:**
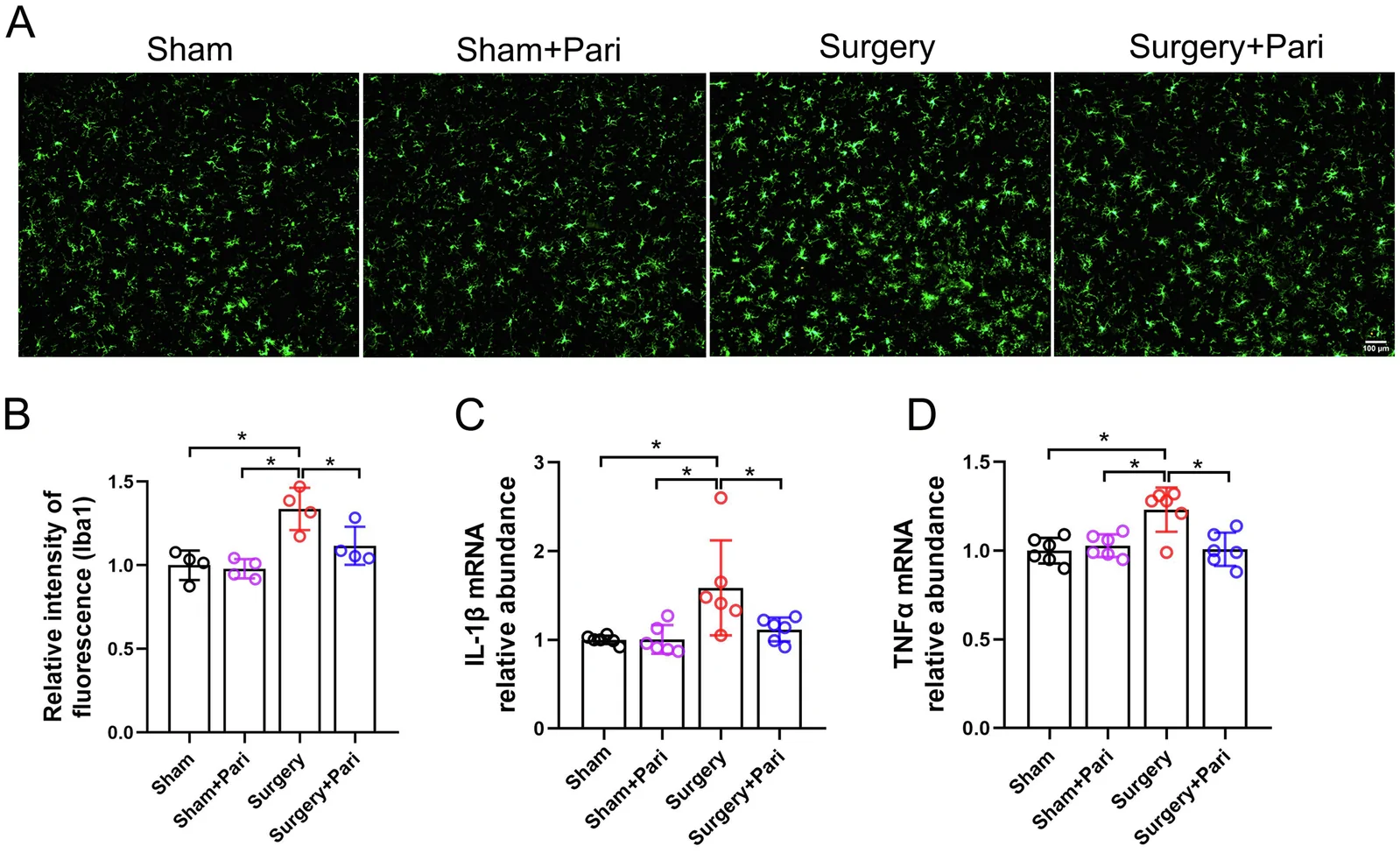
Paricalcitol inhibited hippocampal neuroinflammation in CPTP rats. (A) Representative images of Iba1 staining in the molecular layer of the dentate gyrus (MoDG). Scale bar = 100 μm. (B) Relative fluorescence intensity of Iba1. *n* = 4 rats per group. (C) *IL-1β* mRNA relative abundance. *n* = 6 rats per group. (D) *TNF-α* mRNA relative abundance. *n* = 6 rats per group. Data are expressed as the mean ± SD. *p < 0.05 for Surgery versus Sham, Sham+Pari or Surgery+Pari in B–D.

### 3.4 Paricalcitol rescued gut microbiota dysbiosis and colonic VDR reduction in CPTP rats

Several studies have demonstrated a close association between vitamin D and gut microbiota, wherein vitamin D modulates gut microbial composition primarily via regulation of VDR status (Cantorna et al., 2014). We therefore investigated the gut microbiota composition in Sham, Surgery, and Surgery+Pari groups using 16S rDNA sequencing. Alpha-diversity assessed by Chao (*p* = 0.8595) and Ace (*p* = 0.8840) indices revealed no intergroup differences (Fig. 4A, B).Partial least squares discriminant (PLS-DA) analysis were performed to compare the gut microbial profiles. Samples from the Sham, Surgery and Surgery+Pari groups showed distinct clustering patterns, indicating divergent gut microbial composition in the three groups (Fig. 4C). Next, we examined gut-microbiota differences across distinct taxonomic levels. As illustrated in Fig. 4D and E, gut microbial community composition in each group was profiled at the family and genus levels. At the family level, *Lachnospiraceae* and *Oscillospiraceae* represented the two dominant taxa, collectively accounting for nearly 40% of the total gut microbiota. Notably, the relative abundance of *Lachnospiraceae* was elevated in the Surgery group (29.45%), compared with the Sham group (26.12%) and the Surgery+Pari group (25.36%). While the relative abundance of *Oscillospiraceae* was reduced in the Surgery group (9.39%) compared with the Sham group (16.56%) and the Surgery+Pari group (19.62%). A similar situation was also observed at the genus level. In two of the top three genera, *UCG-005* exhibited lower relative abundance in the Surgery group (4.35%) relative to the Sham group (6.14%) and the Surgery+Pari group (14.68%). By contrast, the relative abundance of *unclassified_f__Lachnospiraceae* was higher in the Surgery group (10.39%) than that in the Sham group (9.56%) and the Surgery+Pari group (5.98%). These suggested that CPTP induced gut microbiota dysbiosis, and paricalcitol partly rescued it. In addition, at the family taxonomic level, LEfSe analysis identified *unclassified_c_Clostridia* as enriched in the Sham group, *Bifidobacteriaceae* enriched in the Surgery group, and *Monoglobaceae* enriched in the Surgery+Pari group (Fig. 4F). At the genus level, LEfSe analysis indicated that the relative abundances of *Prevotellaceae_UCG-001*, *unclassified_c_Clostridia*, *unclassified_f_Peptostreptococcaceae*, and *Papillibacter* were higher in the Sham group compared with the remaining experimental groups. By contrast, *Bifidobacterium*, *Faecalibaculum*, and *Dubosiella* exhibited greater relative abundances in the Surgery group relative to the other groups. The relative abundances of Monoglobus, Chloroplast, Gordonibacter, and Facklamia were higher in the Surgery+Pari group compared with the other groups (Fig. 4G). The Kruskal-Wallis H test was applied for intergroup comparisons. The top 15 most differentially abundant taxa at the family and genus levels were presented in Fig. 4H and I, respectively. Collectively, these findings indicated that gut microbial community composition was markedly altered in Surgery rats relative to Sham animals, and paricalcitol could partially reverse such dysbiosis in Surgery+Pari rats (Fig. 4H and I).

**Fig. 4:**
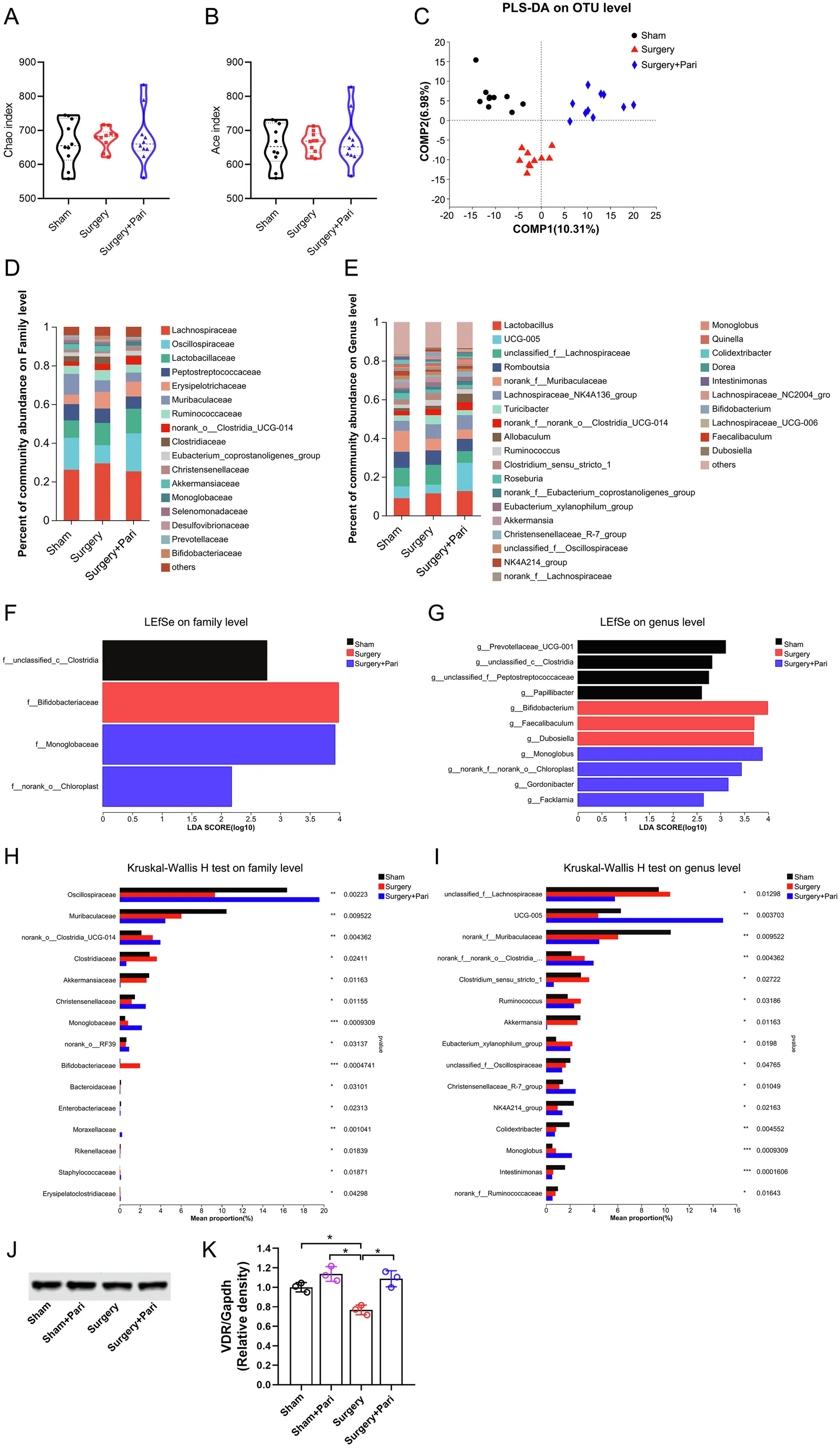
Paricalcitol rescued gut microbiota dysbiosis and colonic VDR reduction in CPTP rats. (A) Alpha-diversity analysis based on the Chao index. (B) Alpha-diversity analysis based on the Ace index. (C) PLS-DA analysis at the OTU level. (D) Relative community abundance at the family level. (E) Relative community abundance at the genus level. (F) LEfSe analysis at the family level. (G) LEfSe analysis at the genus level. (H) Top 15 most differentially abundant bacteria at the family level. (I) Top 15 most differentially abundant bacteria at the genus level. (J) Representative images of VDR protein expression in the colon. (K) VDR/Gapdh relative density. *n* = 3 rats per group. *p < 0.05.

We further examined colonic VDR protein expression (Fig. 4J). GAPDH-normalized VDR protein levels differed significantly among the four groups [Fig. 4K; one-way ANOVA, F (3, 8) = 18.76, *p* = 0.0006]. Tukey's post-hoc tests revealed that colonic VDR protein expression was lower in the Surgery group relative to both the Sham and Sham+Pari groups (*p* = 0.0105 and 0.0006, respectively). VDR expression was significantly higher in the Surgery+Pari group compared with the Surgery group (*p* = 0.0015). No significant difference was detected between the Sham and Sham+Pari groups (*p* = 0.1241). These findings indicate that paricalcitol attenuates the CPTP-associated reduction in colonic VDR protein expression.

## 4 Discussion

The present study demonstrated that CPTP induced severe cognitive dysfunction in rats. CPTP model animals developed mechanical hypersensitivity, accompanied by impaired hippocampal neuronal plasticity, exaggerated neuroinflammation, gut microbiota dysbiosis, and reduced colonic VDR protein expression. Paricalcitol intervention substantially reversed these pathological phenotypes: it attenuated mechanical pain hypersensitivity, restored hippocampal neuroplasticity, suppressed neuroinflammation, ameliorated gut microbiota dysbiosis, and rescued the surgery-associated downregulation of colonic VDR. These findings suggest that paricalcitol confers protective effects against CPTP-related cognitive deficits, likely through targeting the vitamin D receptor.

Accumulating studies have focused on cognitive dysfunction secondary to chronic pain, a devastating complication that severely impairs patients' quality of life and increases healthcare burdens (Liu et al., 2014; Whitlock et al., 2017). However, most existing work focuses on neuropathic pain, whereas CPTP remains understudied in preclinical models despite its high clinical incidence of 30%–40%. In this context, the present study innovatively characterized CPTP-associated cognitive dysfunction using a well-established rat CPTP model. Behavioral assessments were systematically performed to evaluate learning and memory abilities, with locomotor activity firstly examined to rule out motor dysfunction interference. No significant intergroup differences in locomotor activity were observed, confirming that CPTP does not affect basic motor function in rats. Notably, surgery rats exhibited markedly reduced preference for novel objects in the novel object recognition test and displayed prolonged escape latency alongside increased error counts in the Barnes maze test. These behavioral outcomes are highly consistent with previous findings regarding chronic pain-associated cognitive dysfunction (Hu et al., 2010). More importantly, paricalcitol intervention effectively rescued CPTP-associated cognitive dysfunction in rats, preliminarily verifying the protective efficacy of paricalcitol against CPTP-associated cognitive dysfunction.

To further elucidate the neural mechanisms underlying CPTP-associated cognitive dysfunction, we subsequently detected alterations in hippocampal neuroplasticity across experimental groups. Accumulating evidence has demonstrated that hippocampal neurogenesis is critically involved in the regulation of cognitive function, and its dysfunction contributes substantially to cognitive impairment (Toda et al., 2019; Leiter et al., 2022). Consistent with these reports, our results confirmed suppressed hippocampal neurogenesis in CPTP model rats, and this pathological alteration was partially restored by paricalcitol administration. In addition, NMDA and AMPA receptors are essential for maintaining normal neuronal function, and their dysfunction is tightly associated with various cognitive disorders, including Alzheimer's disease and multiple neuropsychiatric diseases (Babaei, 2021). Accordingly, we further detected the expression of NMDA receptor subunits (NR1, NR2A, NR2B) and AMPA receptor subunits (GluA1, GluA2). CPTP specifically downregulated the expression of NR2B, while no significant changes were observed in the levels of NR1, NR2A, GluA1, and GluA2. Notably, paricalcitol treatment effectively reversed the CPTP-induced reduction in NR2B expression. Collectively, these findings suggest that paricalcitol ameliorates CPTP-associated cognitive dysfunction potentially by restoring impaired hippocampal neurogenesis and normalizing NR2B expression.

Numerous preclinical studies have demonstrated that chronic pain triggers robust cerebral neuroinflammation (Dai et al., 2019), which perturbs neuronal function and ultimately promotes cognitive impairment (Li et al., 2020). The vitamin D receptor (VDR) exerts a pivotal function in inflammatory regulation (Shi et al., 2022) and participates in the modulation of neuroinflammatory responses (Pepineli et al., 2019). To dissect the mechanisms driving CPTP-associated cognitive dysfunction, we assessed hippocampal neuroinflammation in this model. In line with prior reports, CPTP elicited marked neuroinflammation. Paricalcitol treatment alleviated CPTP-associated cognitive dysfunction and concurrently attenuated hippocampal neuroinflammation. These findings imply that suppression of CPTP-evoked neuroinflammation may represent one mechanism through which paricalcitol improves CPTP-related cognitive dysfunction.

In order to investigate a potential link between intraperitoneal injection of paricalcitol and neuroinflammation in the brain, we examined the expression of VDR in the abdominal colon. In this study, the expression of VDR was significantly decreased in the Surgery rats, and this decrease was restored after injection of paricalcitol. The activation of VDR by paricalcitol was consistent with a previous study (Hu et al., 2020). Interestingly, a close biological connection between VDR activity and gut microbiota composition has been reported (Cantorna et al., 2014). Intestinal epithelial VDR deficiency can alter microbial assemblage and exacerbate inflammatory responses (Wu et al., 2020). Furthermore, genome-wide association analyses have confirmed that VDR genetic variation is a key determinant shaping gut microbiome profiles (Wang et al., 2016). Consistently, multiple studies have demonstrated that VDR activation via vitamin D3 or 1,25(OH)₂D₃ supplementation increases the abundance of beneficial microbes and effectively alleviates gut microbiota dysbiosis (Ooi et al., 2013). In the current study, alpha diversity did not differ significantly across groups, indicating unaltered microbial richness and diversity among rats. However, PLS-DA revealed distinct microbial clustering patterns among the Sham, Surgery, and Surgery+Paricalcitol groups. In line with this finding, comparisons of gut microbiota composition at both the family and genus levels demonstrated that the dominant taxa in the Surgery group differed from those in the Sham and Surgery+Paricalcitol groups. These data indicate that CPTP induces gut microbiota dysbiosis without altering overall microbial richness or diversity, and paricalcitol ameliorates this compositional imbalance to a certain degree. Using LEfSe analysis, we identified taxa with differential abundance and screened 15 representative bacterial taxa at the family and genus levels, which may serve as candidate biomarkers for CPTP-associated cognitive dysfunction. Furthermore, our results suggest that CPTP downregulates colonic VDR expression, whereas paricalcitol treatment restores colonic VDR expression and partially reverses CPTP-associated shifts in microbial composition.

Our study has several limitations. First, we cannot establish definitive causal relationships among CPTP-associated cognitive dysfunction, neuroinflammation, and gut microbiota dysbiosis. In the absence of microbiota-targeted interventions, it remains difficult to dissect the specific contribution of microbial dysbiosis to cognitive deficits. Second, only male rats were included, which restricts the generalizability of our findings to female animals. Third, paricalcitol ameliorated both cognitive dysfunction and chronic postoperative pain; accordingly, we cannot distinguish whether its cognitive protection stems directly from central mechanisms or is secondary to pain relief. Fourth, cognitive behavioral assessments were only performed at a late postoperative time point, with no testing on postoperative days 7 and 14. This precludes full temporal characterization of the onset and peak severity of CPTP-associated cognitive dysfunction. Fifth, the microbiota analyses lacked a Sham+Paricalcitol group, so we were unable to evaluate whether paricalcitol alone alters gut microbial composition in sham-operated rats. Finally, we did not quantify hippocampal VDR expression and activity. This limitation prevents us from delineating the crosstalk between peripheral and central VDR signaling, and we cannot definitively confirm a direct central action of paricalcitol in the hippocampus. Taken together, although paricalcitol improved CPTP-associated cognitive dysfunction in this rat model, the precise mechanisms mediating this protective effect require further validation.

Despite these limitations, to our knowledge, this is the first study to characterize CPTP-associated cognitive dysfunction and demonstrate that paricalcitol attenuates such cognitive dysfunction in this rat model. CPTP induces impaired hippocampal neurogenesis, reduced NR2B expression, excessive neuroinflammation, gut microbiota dysbiosis, and downregulation of colonic VDR protein expression, while paricalcitol ameliorates these pathological alterations and restores colonic VDR levels. Previous studies has linked gut microbiota dysbiosis to neuroinflammation, suggesting that microbiota-targeted interventions can dampen neuroinflammatory responses (Wang et al., 2021). Collectively, our findings raise the possibility that VDR restoration by paricalcitol confers cognitive protection potentially via the gut microbiota–neuroinflammation–neuroplasticity axis, although this mechanistic cascade requires further validation. These results provide preclinical evidence supporting paricalcitol as a promising candidate for the treatment of CPTP-associated cognitive dysfunction.

## 5 Conclusions

Paricalcitol ameliorates CPTP-associated cognitive dysfunction in rats. CPTP induces impaired hippocampal neuroplasticity, neuroinflammation, gut microbiota dysbiosis, and downregulation of colonic VDR protein expression, whereas paricalcitol mitigates these pathological changes and restores colonic VDR levels. Our findings suggest that paricalcitol-mediated VDR restoration may confer cognitive protection via the gut microbiota–neuroinflammation–neuroplasticity axis, providing preclinical evidence supporting paricalcitol as a therapeutic candidate for chronic postoperative pain-related cognitive dysfunction.

## CRediT authorship contribution statement

**Ning Zheng:** Writing – original draft, Visualization, Validation, Software, Resources, Project administration, Methodology, Investigation, Data curation, Conceptualization. **Yufeng Zhang:** Writing – original draft, Validation, Software, Resources, Methodology, Investigation, Formal analysis, Data curation, Conceptualization. **Cancan Cheng:** Validation, Project administration, Methodology, Investigation, Formal analysis. **Munle Chin:** Software, Project administration, Methodology, Investigation. **Wenxiang Qing:** Writing – review & editing, Writing – original draft, Visualization, Validation, Supervision, Software, Resources, Project administration, Methodology, Investigation, Funding acquisition, Formal analysis, Data curation, Conceptualization. **Qin Liao:** Writing – review & editing, Writing – original draft, Visualization, Validation, Supervision, Software, Resources, Project administration, Methodology, Investigation, Funding acquisition, Formal analysis, Data curation, Conceptualization.

## Ethics declaration

This study was conducted in accordance with the ARRIVE (Animal Research: Reporting of In Vivo Experiments) guidelines. This study was approved by the Animal Ethics Committee of the Central South University. (Approval No. CSU-2022-0001-0312)

## Funding

This study has been supported in part by 10.13039/501100001809National Natural Science Foundation of China (No. 82301381, 82571455) and Health Research Project of Hunan Provincial Health Commission (20256849).

## Declaration of competing interest

The authors declare that they have no known competing financial interests or personal relationships that could have appeared to influence the work reported in this paper.

## Data Availability

Data will be made available on request.
